# Exploring Rurality and Racial/Ethnic Disparities in Texas Mortality: A Scoping Review of the Five Leading Causes of Death

**DOI:** 10.1007/s40615-025-02627-y

**Published:** 2025-09-17

**Authors:** Gogoal Falia, Emesomhi Eboreime, Peter Harper, Amy Willa, Alva O. Ferdinand

**Affiliations:** 1 School of Public Health, Texas A&M University, College Station, TX 77843 USA; 2 Southwest Rural Health Research Center, College Station, TX 77843 USA; 3 Iowa Primary Care Association, Des Moines, IA 50309 USA; 4 Episcopal Health Foundation, Houston, TX 77002 USA; 5 Department of Health Policy & Management, Texas A&M University School of Public Health, 212 Adriance Lab Rd, Suite 160, TAMU 1266, College Station, TX 77843-1266 USA

**Keywords:** Ethnic and racial minorities, Cause of death, Intersectional framework, Rural population, Review, Community health

## Abstract

**Objective:**

While the five leading causes of death in Texas mirror national trends, the unique racial/ethnic and geographical diversity of the state requires a focused analysis of mortality disparities. To address this gap, we conducted a scoping review of Texas-specific peer-reviewed studies on how race/ethnicity and rurality impact mortality from heart disease, cancer, unintentional injuries, stroke, and chronic lower respiratory diseases (CLRDs).

**Methods:**

Using a scoping review methodology, we identified Texas-specific empirical articles examining the relationship between either rurality or race/ethnicity and the leading causes of death. We extracted and analyzed data from the included studies using a coding instrument specifically designed for this study. Descriptive synthesis summarized study trends, and multivariable logistic regression assessed overarching patterns.

**Results:**

Our synthesis of the Texas-specific literature reveals that cancer was the focus of nearly half of the studies (49.4%), whereas rurality was seldom examined (4.2%). Multivariable logistic regression showed that studies on CLRDs were more likely to find higher mortality among Blacks (*OR* = 3.07) and Hispanics (*OR* = 4.24). For stroke, Hispanics were associated with increased mortality (*OR* = 2.49), while for unintentional injuries, the study found significantly higher mortality for Native Americans (*OR* = 8.72) and Blacks (*OR* = 2.76).

**Conclusions:**

Existing Texas-focused studies consistently link racial and ethnic minority status with higher mortality from the five leading causes of death. However, significant gaps remain, particularly concerning the intersection of race/ethnicity and rurality. Future Texas-based research should prioritize these intersectional factors to better understand and address persistent mortality disparities.

## Introduction

Heart disease, cancer, unintentional injuries, stroke, and chronic lower respiratory diseases (CLRDs) have been the five leading causes of death in the USA for over a decade [1]. Data from the Centers for Disease Control and Prevention (CDC) indicated that over 1.8 million people died from these five diseases in 2022 [2]. Death rates per 100,000 populations were 167.2 for heart disease, 142.3 for cancer, 64.0 for accidents, 39.5 for stroke, and 34.3 for CLRD [2]. The USA currently has an annual healthcare expenditure of approximately $4.5 trillion, with 90% allocated to chronic conditions [3]. Yearly productivity losses due to heart disease and premature mortality were reported to be $210 billion in 2020, while the same for fatal unintentional injuries was $69 billion [4, 5]. Similarly, the economic burden from mortality-related loss of earnings from cancer and stroke has been reported to be more than $94 billion and $30 billion, respectively [6–8]. Further, CLRD-related mortality has been linked to almost $50 billion in productivity losses [9]. Socioeconomic burdens of these diseases call for more attention to the underlying factors that contribute to these conditions and the mortality burden that they present.

Several studies have documented disparities in mortality burdens from these diseases by race/ethnicity and across urban and rural areas [10]. In contrast to urban residents, rural dwellers experience higher poverty levels, lower educational attainment, higher levels of transportation challenges, are more elderly on average, and have limited healthcare access, leading to elevated mortality rates for leading causes of death across all racial/ethnic groups [11–13]. Studies further underscore that rural residents are more likely than their urban counterparts to live with or die from one of the five leading causes of death [14–24]. Researchers have also noted racial/ethnic disparities in disease prevalence and mortality in studies examining the leading causes of death individually. For instance, prior studies have shown that African Americans experience higher rates of cardiovascular disease risk factors, such as hypertension, diabetes, and obesity, compared to non-Hispanic Whites, contributing to elevated cardiovascular-related mortality in this group [13, 15, 19]. Further, African Americans face higher mortality rates for cancer and stroke, compared to other racial/ethnic groups [13, 21]. Cancer is the leading cause of death among Hispanics, accounting for 21% of adult deaths attributed to later-stage diagnoses in Hispanic people [16, 25]. Mortality from unintentional injury and chronic lower respiratory diseases is disproportionately higher for American Indian/Alaskan Native and African American populations [13, 15, 23, 24, 26–28].

According to the U.S. Census Bureau, Texas has the largest rural population of the 50 states, with over 4,740,000 rural residents [29]. Moreover, as of 2020, Texas is among the top 10 most diverse states in the USA, with a 67% chance that two people chosen at random will be from different racial and ethnic groups [30]. This percentage is higher than the national average of 61% [30]. Importantly, the five leading causes of death in Texas are the same as those at the national level, with Texas residents experiencing elevated mortality rates from heart disease (172.3 per 100,000 population) and stroke (42.3 per 100,000), compared to the national average (167.2 per 100,000 population and 39.5 per 100,000 population) in 2022 [2, 31]. In 2023, cancer was the second leading cause of death in Texas, accounting for more than 50,908 deaths statewide [32, 33]. Consistent with national trends, Texas exhibits persistent health disparities. Multi-state studies have found greater prevalence of cancer and heart disease mortality among African Americans in Texas linked to various factors, such as high rates of uninsurance, socioeconomic deprivation, prevalence of chronic diseases, and a significant rural population [33–35]. While previous research has broadly examined the five leading causes of death and their associations with race/ethnicity and rurality, these studies have largely been multistate or national, with limited findings specific to Texas. Additionally, few reviews have addressed these causes collectively, hindering public health action at the state level. Understanding where gaps lie is especially important in Texas, where an estimated 4.9 million people remained uninsured in 2023 (1.75 times the national average), and where 235 of the state’s 254 counties face healthcare professional shortages [36].

Therefore, the purpose of this study was to conduct a scoping review of Texas-specific literature examining the five leading causes of death. We were particularly interested in the extent to which studies included measures of race/ethnicity and rurality in their analyses and whether these factors were found to have significant relationships with the five leading causes of death. Our work supplements previous reviews that narrowly focused on the cost-effectiveness of interventions aimed at any of the five leading causes of death [37], health behaviors associated with the five leading causes of death [38], and reviews done on leading causes of death independently [39–42]. Overall, our study helped identify gaps in the literature about the unique demographic landscape in Texas as it relates to the five leading causes of death, providing a useful synthesis of the evidence for stakeholders like healthcare and public health professionals, providers, policymakers, insurers, and philanthropic organizations, to use in their efforts to improve healthcare outcomes in the state.

## Methods

**Fig. 1:**
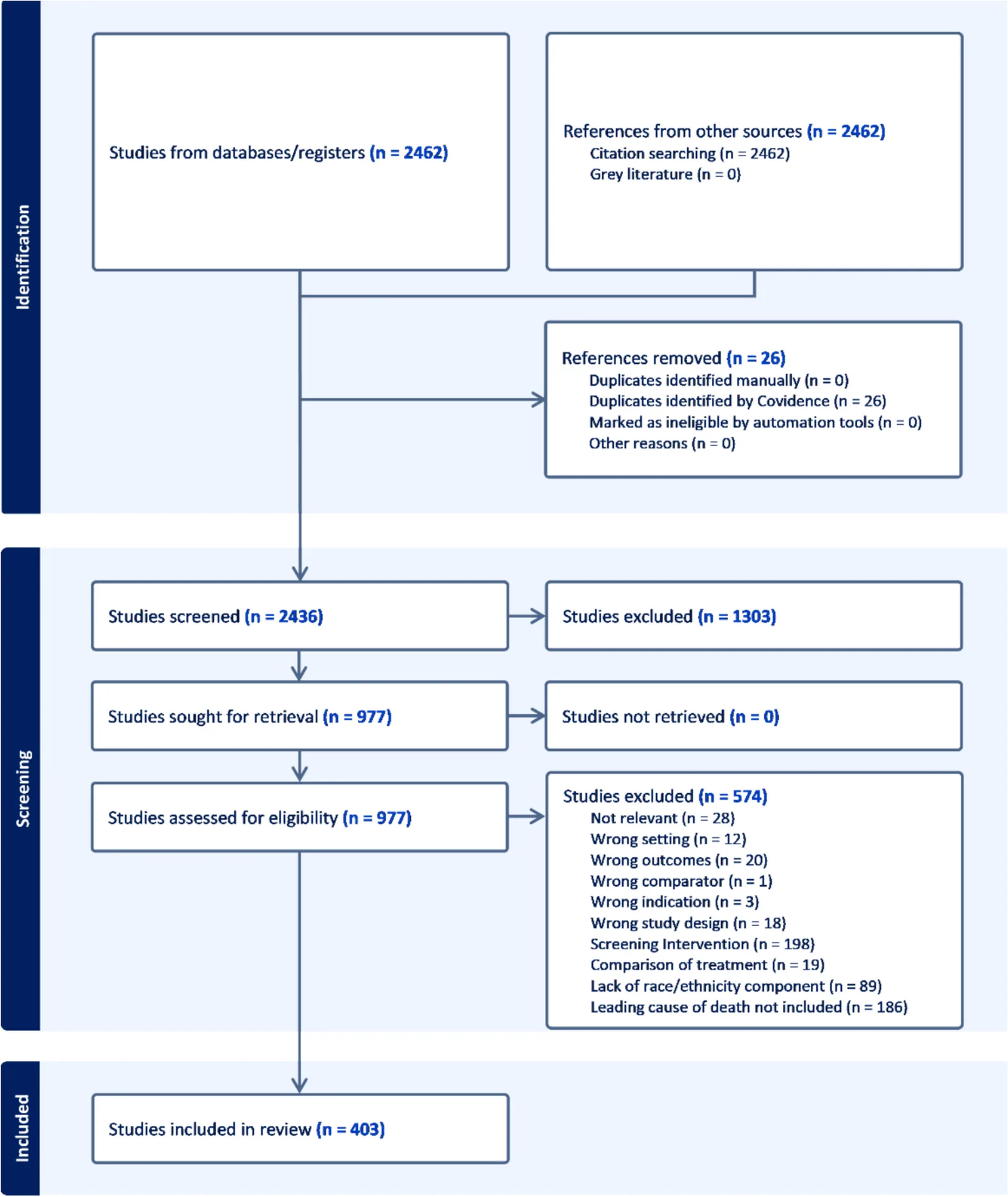
Preferred Reporting Items for Systematic Reviews and Meta-analyses (PRISMA) flow diagram summarizing the literature search, screening, and selection process

We used a scoping review methodology to identify and extract information from articles published on the five leading causes of death in Texas. Figure 1 depicts the search process following the Preferred Reporting Items for Systematic Reviews and Meta-analyses (PRISMA) guidelines [43]. We included articles that were published through October 2022. We enlisted the assistance of a professionally trained systematic and scoping review librarian from the Texas A&M University Libraries. We searched the MEDLINE and CINAHL Plus databases for relevant literature. A preliminary title review was conducted to identify studies that mentioned race/ethnicity, rurality, Texas, and at least one of the five leading causes of death. There were 2462 articles identified for title screening. Titles were excluded from further review if they were systematic reviews, commentaries, reports, or summaries. After determining which articles should be excluded after title review, study abstracts were reviewed. The goal of the abstract review process was to identify a leading cause of death under study and the inclusion of rurality or race/ethnicity as an independent variable. Abstracts were rejected if they were qualitative studies, a comparison of treatments for a leading cause of death, lacked a rural, racial, and ethnic minority component, or did not focus on the top five leading causes of death. In this step, 977 articles met our criteria of studying a leading cause of death and included rural residence or racial/ethnic minority status as independent variables. The full texts of these 977 articles were then reviewed with a focus on a high level of specificity. Articles that did not include rural residence or racial/ethnic minority status in their studies of a leading cause of death were excluded. Disagreements about inclusion or exclusion were reconciled by group discussions among the authors. We identified 403 full-text articles for final inclusion in this scoping review.

We systematically classified all included articles by using a pre-determined extraction tool and the Covidence platform [44]. From each article, we collected information on the leading cause of death studied, the first author’s affiliation, the year the study was published, the extent to which the study was conducted exclusively in Texas, in Texas, and a handful of other states, or was national in scope with specific reference to Texas-based results. We additionally extracted information on the dataset used to explore the leading cause of death in question, as well as the type of rurality measure used. Importantly, we extracted information on whether race/ethnicity status or rural residence was associated with higher mortality or morbidity with respect to the leading cause of death studied.

First, we conducted descriptive analyses to examine the distributions of extracted information. Additionally, we explored the trends in the publication of papers on the five leading causes of death in 10-year increments to determine whether some causes of death have been understudied with respect to rurality and racial/ethnic minority status. Key study characteristics were also extracted, including first author affiliation (Academic Medical Center, University Department, and Others), journal type (General Journal and Specialty Journal), study location (Texas Only, Texas and Other Areas, Texas County/Zip Code, and Nationwide), and age-group studied (0–17 years, 18–64 years, 65 years and above, and ‘Others’ coded for zero to end-of-life).

We classified the datasets used as the Texas Cancer Registry, Texas State Health Databases (Texas Department of State Health Services [DSHS], the Texas Death Registry, State Inpatient Databases [SID], and the Texas Emergency Medical Services [EMS)/Trauma Registry]), National Databases (Surveillance, Epidemiology, and End Results [SEER] and Behavioral Risk Factor Surveillance System [BRFSS]), and Others (Dallas Heart Study, San Antonio Heart Study, Corpus Christi Heart Project, the Brain Attack Surveillance in Corpus Christi [BASIC] Project, the Texas Hospital Inpatient Discharge Public Use Data File, Hispanic Established Population for the Epidemiological Study of the Elderly [Hispanic EPESE], and medical and discharge records from university hospital centers across Texas). Measure of rurality was classified into established codes such as Rural–Urban Continuum Codes (RUCC), and Rural–Urban Commuting Area (RUCA), while we used “Others” for a large portion of rurality, defined using non-standardized, custom definitions on population density or through simple urban–rural labeling. We used “no clear measurement” to include articles where rural populations were either not included or were not clearly defined.

We calculated the number of studies on the five leading causes of death, which included rurality and racial/ethnic minority status as key predictor variables. These variables were classified into race/ethnicity, rurality, and both race/ethnicity and rurality. Similarly, observed association with higher mortality was classified as race/ethnicity, rurality, and both race/ethnicity. We also included a breakdown of race/ethnicity for association with higher mortality and categorized it as Black, Hispanic, Native American, Asian/Pacific Islander, and Others.

Lastly, we conducted multivariable logistic regression using a stepwise method (with *p*-value threshold of 0.05 for entry and 0.10 for removal) to assess whether studies reported higher mortality rates among individuals from rural or racial/ethnic minority backgrounds for the five leading causes of death in Texas. Analyses were reported as adjusted odds ratios (*OR*s) with robust standard errors (*SE*), and performed in Stata 15.0 MP (College Station, TX, USA). Statistical significance was indicated at *p* < 0.05.

## Results

**Fig. 2:**
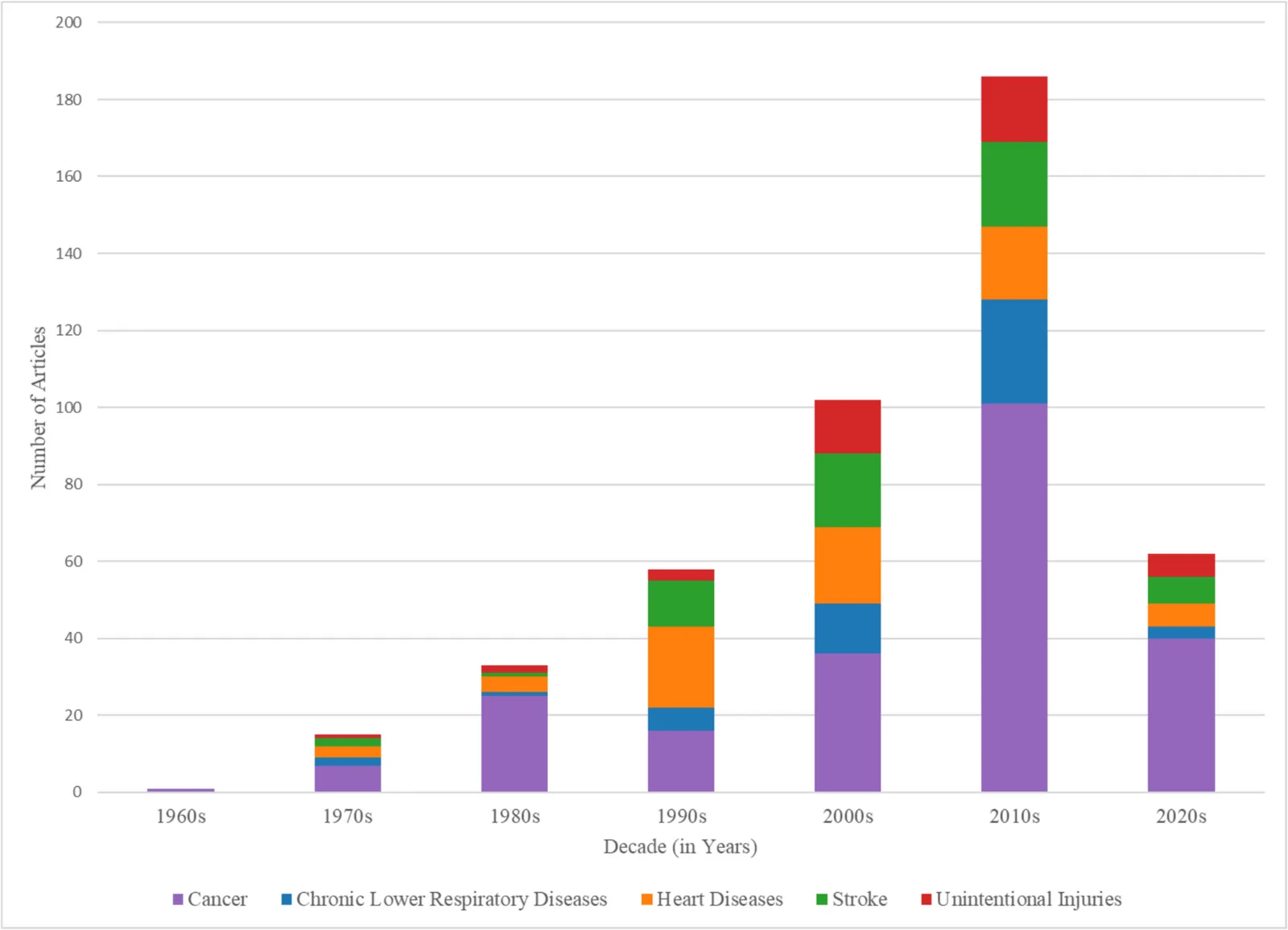
Evolution of papers on the five leading causes of death over time that account for race/ethnicity and rurality in Texas (1960–2022)

The sample contained 457 analyses clustered within 403 articles that met our criteria. The number of published articles examining the five leading causes of death with race/ethnicity or rurality included as potential contributing factors increased over time from 1 paper in the 1960 s to 62 papers in the 2020 s (Fig. 2).

The highest number of publications during the study period was observed in 2017 (*N* = 28). Our findings show that from the 1970 s to 2022, there was a steady increase in the number of articles published overall. Nevertheless, articles on cancer that included race/ethnicity and race as potentially contributing factors saw a substantial jump between the early 2000 s and 2022. Comparatively, the number of articles on the other leading causes of death (heart disease, unintentional injuries, stroke, and CLRDs) that accounted for race/ethnicity and rurality rose minimally relative to articles on cancer during our study period.

Descriptive characteristics of the studies are presented in Table 1. We found race/ethnicity and rurality were associated with mortality in 230 (50.4%) and 19 (4.2%) articles, respectively. Of the articles that accounted for either rurality or race/ethnicity in their study of a leading cause of death, cancer was studied in almost half of the included articles (*N* = 226; 49.4%). Many of the leading authors of these articles were affiliated with academic medical centers (*N* = 203; 44.4%), while others were from non-medical university departments (*N* = 183; 40.0%) or outside of academia entirely (e.g., governmental agencies) (*N* = 71; 15.5%). Most of the articles were published in specialty journals (*N* = 351; 76.8%) and were based exclusively in Texas (*N* = 375; 82.0%). Studies accounting for the adult age group (18 to 64 years old) were the most frequently studied (*N* = 302; 66.1%). Researchers frequently used the Texas Cancer Registry (*N* = 95; 20.8%) and the Texas State Health Databases (*N* = 53; 11.6%) to study the leading causes of death. A greater proportion of the dataset fell under the “Others” category, comprising 266 cases (58.2%). As shown in Table 1, a significant majority of studies are classified as having “No clear measurement,” encompassing articles where rural populations are either not included or are not clearly defined (*N* = 371; 81.2%). Similarly, few studies used standardized coding systems such as RUCC (*N* = 9; 2.0%) or RUCA (*N* = 10; 2.2%) codes, while a large portion defined rurality using non-standardized, custom definitions on population density or through simple urban–rural labeling. This “Other” group comprised 67 studies (14.7%). Our analysis of the primary disparity factors revealed that race/ethnicity was a frequently used predictor (*N* = 338; 74.0%), whereas rurality was included far less often (*N* = 34; 7.4%), and never as a standalone factor. Ultimately, just over half of all studies (*N* = 230; 50.4%) documented a significant association between race/ethnicity and higher mortality, with Hispanic (*N* = 171; 37.4%) and Black (*N* = 121; 26.5%) populations most frequently identified.

**Table 1:** Descriptive statistics of articles reviewed (*N* = 403) on rurality and race/ethnicity as they relate to the five leading causes of death in Texas

Article characteristic	*N* (%)
Leading cause of death	
Cancer	226 (49.4)
Stroke	63 (13.8)
Heart disease	73 (16.0)
Chronic lower respiratory diseases (CLRDs)	52 (11.4)
Unintentional injury	43 (9.4)
First author affiliation	
Academic Medical Center	203 (44.4)
University Department	183 (40.0)
Others	71 (15.5)
Journal type	
General journal	106 (23.2)
Specialty journal	351 (76.8)
Year published	
1960–1969	1 (0.2)
1970–1979	15 (3.3)
1980–1989	33 (7.2)
1990–1999	58 (12.7)
2000–2009	102 (22.3)
2010–2019	186 (40.7)
2020–2022	62 (13.6)
Study location	
Texas only	273 (59.7)
Texas and other areas	78 (17.1)
Texas county/zip code	102 (22.3)
Nationwide	4 (0.9)
Age group studied	
Pediatric (0 to 17)	68 (14.9)
Adult (18–64)	302 (66.1)
Elderly (65 and older)	48 (10.5)
Others	39 (8.5)
Dataset used	
Texas Cancer Registry	95 (20.8)
Texas State Health Databases	53 (11.6)
National databases	21 (4.6)
Others	288 (63.0)
Rurality measurement used	
Rural–Urban Continuum Codes (RUCC)	9 (2.0)
Rural–Urban Commuting Area (RUCA) codes	10 (2.2)
Others	67 (14.7)
No clear measurement	371 (81.2)
Predictor variables examined in Texas studies	
Race/ethnicity	338 (74.0)
Rurality	0 (0.0)
Race/ethnicity and rurality	34 (7.4)
Observed association with mortality in Texas studies^1^	
Race/ethnicity	230 (50.4)
Breakdown by racial/ethnic group	
Hispanic	171 (37.4)
Black	121 (26.5)
Native American	4 (0.9)
Asian/Pacific Islander	6 (1.3)
Others	45 (9.9)
Rurality	19 (4.2)
Race/ethnicity and rurality	13 (2.8)

The final sample contains 457 analyses derived from 403 unique articles.

^1^Categories for the racial/ethnic groups’ breakdown are not mutually exclusive. A single study may report disparities for more than one group.

The results presented in Table 2 depict findings from multivariable logistic regressions. In studies focused on CLRDs, after adjustment for age, study region, and rural characteristics, Black (*OR* = 3.07, *p* < 0.05) race and Hispanic (*OR* = 4.24, *p* < 0.05) ethnicity were associated with higher mortality, respectively. Similarly, Hispanic ethnicity was associated with higher mortality in studies that focused on stroke (*OR* = 2.49, *p* < 0.05). Interestingly, studies on unintentional injuries found that Native American (*OR* = 8.72, *p* < 0.05) and Black (*OR* = 2.76, *p* < 0.05) races were associated with higher mortality, respectively. Race/ethnicity was not identified as a statistically significant predictor in studies examining heart disease and cancer among Texans. Additionally, research that focused on rurality as a predictor of mortality did not yield statistically significant findings.

**Table 2:** Multivariable logistic regression results for the five leading causes of death and race/ethnicity

	**Odds ratio**
Studies focused on chronic lower respiratory diseases (CLRDs)	
Studies where Hispanic ethnicity is associated with higher mortality	4.24 (2.57)
Studies where Black race is associated with higher mortality	3.07 (1.78)
Studies focused on stroke	
Studies where Hispanic ethnicity is associated with higher mortality	2.49 (0.82)
Studies focused on unintentional injury	
Studies where Native American race is associated with higher mortality^1^	8.72 (8.96)
Studies where Black race is associated with higher mortality	2.76 (1.04)

Multivariable logistic regression results with robust standard errors in parentheses. Only statistically significant results (*p* < 0.05) are reported.

^1^Native American findings are based on limited data and must be interpreted with caution.

## Discussion

In this study, we utilized a scoping review methodology to explore the evidence base on the association of rurality and racial/ethnic minority status on the five leading causes of death in Texas. We assessed peer-reviewed articles that examined the five leading causes of death published with Texas-specific data or samples in the past six decades (1960–2022). This literature has grown in recent years, and our work aimed to provide synthesized evidence that can be used to inform strategies for addressing unequal experiences of the five leading causes of death in Texas.

We noted three main findings. First, we found race/ethnicity and rurality were associated with mortality in 230 (50.4%) and 19 (4.2%) articles, respectively. Previous national and Texas-based research has highlighted African American, American Indian/Alaskan Native, and Hispanic populations experiencing higher mortality rates across the five leading causes of death [13, 15, 16, 19, 23–25, 33, 35]. Nearly half of the reviewed articles focused on cancer, likely reflecting the substantial funding and investment in cancer research in the past decade [45]. The presence of M.D. Anderson Cancer Center, a leading institution in cancer treatment and research, also contributed to the number of studies coming out of Texas. However, despite significant overall investment in cancer research, funding remains inequitably distributed, with cancers that disproportionately affect racial and ethnic minorities receiving significantly less support [46, 47]. This disparity skews the research landscape towards cancers more prevalent in other groups, leaving those affecting minority populations understudied and exacerbating existing health inequities. Meanwhile, other leading causes of death-including heart disease, stroke, CLRDs, and unintentional injuries, despite accounting for a substantially higher number of deaths and greater economic impact, remain underrepresented in research, compared to the extensive focus on cancer [1, 4, 5, 7, 8, 48].

Second, our multivariable logistic regressions indicate higher mortality among racial and ethnic minority groups in studies focused on CLRDs. Several researchers report similar patterns, particularly among African American and Hispanic populations [49, 50]. However, other studies find no significant racial or ethnic disparities in CLRD mortality [51, 52]. Notably, access to health insurance, education, and higher income appear to have protective effects against CLRDs among marginalized populations [53, 54]. Our findings from Texas-based studies on stroke mortality show associations primarily with Hispanic ethnicity. This differs from national studies that link both African American race and Hispanic ethnicity to increased stroke mortality [55–57]. Given Texas’ size and demographic diversity, this state-specific pattern underscores the need for more focused research on stroke outcomes among Hispanic populations in Texas. Perhaps most notably, our analysis of unintentional injury mortality in Texas aligns with national evidence showing elevated rates among both Hispanic and Native American individuals [23, 26–28]. Although few Texas-based studies specifically examine unintentional injuries among Native Americans, national findings point to an important gap and opportunity for further research in this area.

Third, our analysis of the relationship between race/ethnicity and leading causes of death revealed no statistically significant associations for heart disease or cancer studies in Texas. These findings diverge from previous studies by Balfour et al. (2016) and Wang et al. (2017), which reported that Hispanic ethnicity was associated with increased mortality risk from heart disease or cancer, respectively [35, 58]. This is despite recent research that has expanded to consider disease burden among racial and ethnic groups in Texas [2, 31, 33, 35]. Similarly, the number of studies that examined rurality as a predictor of mortality was limited and did not show statistical significance. This may be due to a lack of Texas-specific research on the five leading causes of death, with much of the existing literature focused on socioeconomically disadvantaged urban populations with little focus on the rural populations [34, 59].

Rurality did not emerge as a statistically significant predictor of mortality for any of the five leading causes of death in our review. Given the large rural population and uninsurance rates in Texas, this unexpected finding can be due to several factors. Lack of a standardized definition of rurality across studies may have introduced methodological heterogeneity, obscuring true association. Further, the broadness of the five causes of death, each encompassing a wide array of diseases and conditions, could have invariably impacted rurality. While rurality might be a strong predictor for some types of cancers and heart diseases, grouping these leading causes of death can dilute geography-specific effects, leading to an overall non-significant finding [60, 61]. As rurality often acts as a proxy for socioeconomic and access-related factors, these could be the primary driving factors for health disparities. Once controlled for, the effect of rurality can become a non-significant influence on mortality. Contrary to previous national and Texas studies, our findings on race/ethnicity do not emerge as a significant predictor for mortality in Texas-based studies on heart disease and cancer [11–14, 33, 34]. This divergence can be attributed to the high volume of cancer research in the state, which does not fully represent the cancers that disproportionately affect the minority population, already shown to receive less research funding [45–47]. Given the diverse demographic landscape of Texas, the heterogeneity within the state’s racial/ethnic population subgroups can mask health risks and mortality outcomes that are more pronounced in one group compared to another. Ultimately, this review provides evidence on the state of published literature rather than a definitive statement on population health in Texas, highlighting critical gaps, the need for research, and investigation of mortality disparities with an intersectional lens.

Our review of Texas-specific literature on the five leading causes of death reveals critical patterns and significant gaps in the understanding of the roles of race/ethnicity and rurality.

- Research on the five leading causes of death in Texas has overwhelmingly focused on cancer, with studies on the disease (49.4%) accounting for almost as many as studies on the other four leading causes of death combined (50.6%)
- Racial/ethnic disparities in Texas are highly cause-specific, showing significant variations for CLRDs, stroke, and unintentional injuries, whereas no similar association is found for heart disease or cancer.
- Rurality is a critically understudied factor in Texas mortality research, with a majority of studies lacking a clear or standardized measure of rurality.

These findings have direct implications for a variety of stakeholders working to improve health outcomes in Texas. For public health officials, these documented disparities, especially among Hispanics, Blacks, and Native Americans, highlight the urgent need for culturally competent and targeted public health intervention. The lack of research on rural populations signals a major blind spot and requires advocacy and commissioning of research, especially for this population, to investigate their health needs and to ensure equitable resource allocation and program development. For healthcare providers, the association between racial/ethnic minorities and higher mortality for the leading causes of death suggests a need for greater screening, disease management programs, and preventive care support for specific populations. For insurers, this could signal the need for the development of incentives for providers to practice in underserved rural and racial/ethnic minority communities and improve access to care in these areas of need. Lastly, our highlighted research gaps can inform policymakers to develop state-level research funding priorities. Policies should be designed to promote research that examines the intersection of race/ethnicity and geography to create a more complete picture of health disparities in Texas.

## Limitations

While our findings add to the evidence base of health inequities among racial/ethnic and rural populations in Texas, they have a few limitations. First, the review is limited by methodological heterogeneity in how studies measured rurality. An overwhelming majority of the reviewed studies have “No clear measurement” for rurality, where rural populations were either not included or not clearly defined. A significant number of “Other” studies use custom definitions such as population density or simple urban–rural labels without citing a standardized system like RUCC or RUCA. This problem is compounded by the failure of a majority of these studies to specify whether rurality was self-reported or objectively defined by residence. This resulting inconsistency presents a significant barrier to drawing definitive conclusions, as it hampers the comparability of findings across studies, prevents a valid synthesis or pooling of rural health data, and can ultimately mask the true relationship between rurality and mortality in Texas. Second, a limited number of studies specifically focused on the Texas population met our inclusion criteria. Although some studies were national in scope, many state-level analyses grouped Texas with other states, making it difficult to isolate findings unique to the state. Third, small sample size for our multivariable logistic regressions can cause overestimation of the effect [62]. Fourth, there may be publication bias or underreporting of mortality disparities in Texas, especially in rural and underrepresented communities. This could lead to an underestimation of health inequities. Fifth, although our search strategy was developed with an academic librarian, it is possible that our search missed relevant articles across unexplored databases. Our review focused solely on published peer-reviewed literature and did not include grey literature (e.g., government reports and non-peer-reviewed studies), which can provide additional data and insights, particularly from state agencies and public health organizations. Lastly, the review’s focus on Texas can limit the generalizability of its findings. Texas has a distinct demographic landscape, the second most diverse state with the largest rural population, but also has the highest uninsured rates in the USA [29, 30, 63–65]. These state-specific factors likely influence mortality patterns in ways not directly applicable to other states.

Despite the limitations, our findings have some important implications for policy and practice. This study provides an evidence-based synthesis of peer-reviewed literature on the five leading causes of death, specifically as they relate to the burden that rural and racial/ethnic minorities bear in the state of Texas. Our findings indicate critical gaps in the literature and provide the groundwork for future research aimed at examining the intersectionality of rurality and race/ethnicity in shaping mortality patterns across Texas. Given the rapid demographic and economic growth in Texas, there is a pressing need for studies that can further explore how these overlapping social determinants jointly influence access to care and health outcomes.

## Conclusion

This scoping review highlights significant gaps and emerging patterns while examining literature on the five leading causes of death in Texas. While these causes disproportionately affect racial/ethnic minorities, research focus has been disparate, with rurality and intersectional analyses notably underrepresented. Addressing racial/ethnic and geographic inequities is a critical step toward shaping responsive public health efforts.

This review underscores the limitations of current approaches to intersectional research in Texas and provides directions for future studies aimed at addressing these shortcomings. Future research must move beyond a siloed approach and embrace an intersectional framework to untangle complex and overlapping factors that drive health inequities in the state. Methodologically, this requires commitment to using consistent, standardized definitions of rurality, employing longitudinal study designs to track outcomes over time, and applying advanced causal analysis that can effectively analyze complex interactions and mediation effects. Crucially, this approach must prioritize detailed subgroup analyses to better understand how the leading causes of death affect the racial/ethnic population in Texas.

Such evidence-based research will not only document disparities across racial/ethnic and geographical lines but also enable the development and testing of targeted interventions, especially in understudied rural regions. These findings can guide policy development, resource allocation, and community-based initiatives aimed at reducing health inequities. With a sustained commitment to inclusive, evidence-based research, Texas has the opportunity to transform its health landscape and ensure a healthier, more equitable future for all residents.

## Data Availability

The data that support the findings of this study are available upon reasonable request.

## Abbreviations

CDC: Centers for Disease Control and Prevention
PRISMA: Preferred Reporting Items for Systematic Reviews and Meta-analyses
MEDLARS Online (MEDLINE): Medical Literature Analysis and Retrieval System Online
CINAHL: Cumulated Index in Nursing and Allied Health Literature
CLRDs: Chronic lower respiratory diseases
